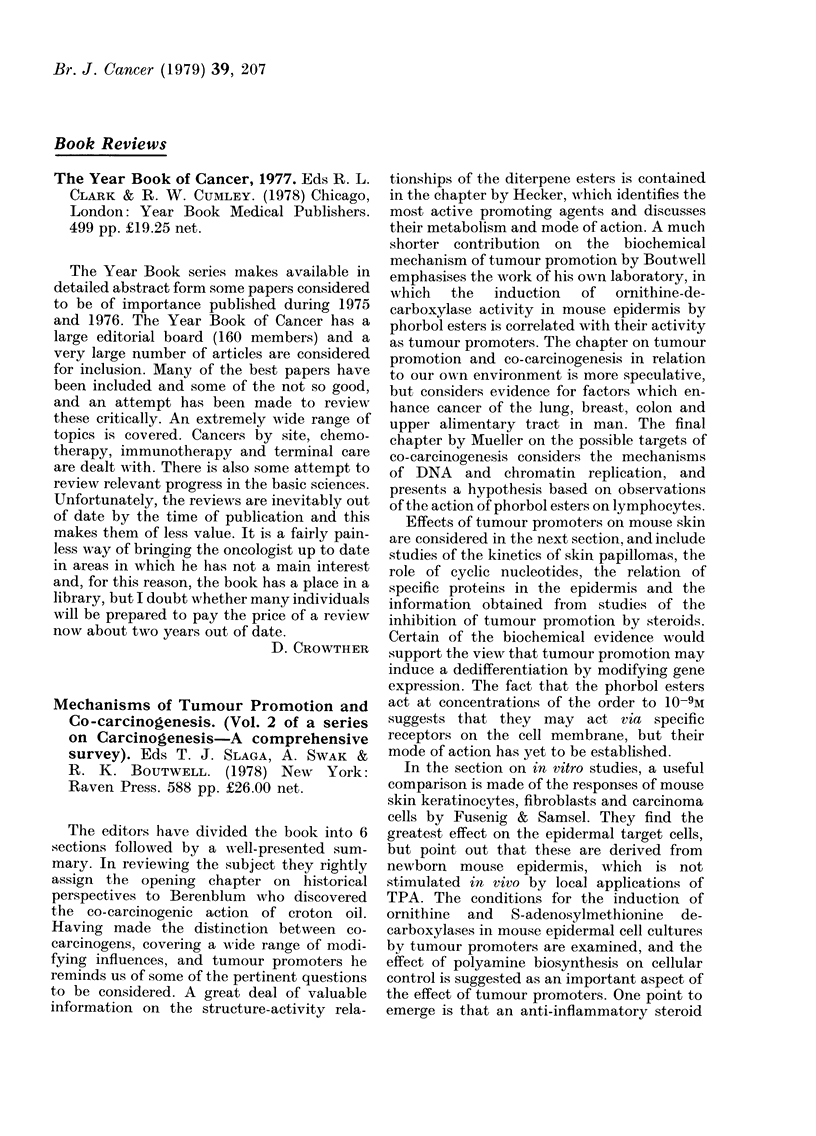# The Year Book of Cancer, 1977

**Published:** 1979-02

**Authors:** D. Crowther


*Br. J. Cancer* (1979) **39**, 207

### *Book Reviews*

**The Year Book of Cancer, 1977.** Eds R. L. Clark & R. W. Cumley. (1978) Chicago, London: Year Book Medical Publishers. 499 pp. £19.25 net.

The Year Book series makes available in detailed abstract form some papers considered to be of importance published during 1975 and 1976. The Year Book of Cancer has a large editorial board (160 members) and a very large number of articles are considered for inclusion. Many of the best papers have been included and some of the not so good, and an attempt has been made to review these critically. An extremely wide range of topics is covered. Cancers by site, chemotherapy, immunotherapy and terminal care are dealt with. There is also some attempt to review relevant progress in the basic sciences. Unfortunately, the reviews are inevitably out of date by the time of publication and this makes them of less value. It is a fairly painless way of bringing the oncologist up to date in areas in which he has not a main interest and, for this reason, the book has a place in a library, but I doubt whether many individuals will be prepared to pay the price of a review now about two years out of date.

D. Crowther